# Serological features of systemic lupus erythematosus diagnosed after referral through a rheumatology triage system because of positive antinuclear antibodies

**DOI:** 10.1186/ar4652

**Published:** 2014-09-18

**Authors:** Marvin J Fritzler, Whitney Steber, Terri Lupton, Christie Fitch-Rogalsky, Liam Martin, Susan G Barr, Ann Clarke

**Affiliations:** 1 University of Calgary, AB Canada

## Background

The initial diagnosis of systemic lupus erythematosus (SLE) is made in a number of clinical settings, which include referrals to a specialist because of a constellation of symptoms and/or abnormal laboratory findings. Although a positive antinuclear antibody (ANA) test has been regarded a serological hallmark of SLE, it is also associated with a number of other systemic autoimmune rheumatic diseases (SARD). We studied the serological features of patients who were referred through a central triage (CT) system because of positive ANA and were then diagnosed as having SLE by the consulting rheumatologist.

## Methods

Patients who met four criteria were included in the SLE cohort: referred to CT over 3 years; reason for referral was positive ANA; evaluated by a certified rheumatologist; and diagnosed as SLE. Clinical information from the first rheumatologic visit was extracted from the consultant's report. An anonymous CT database was developed to contain clinical information and an anonymous serological database was used for autoantibody test results.

## Results

A total of 15,357 patients were referred through the CT; 643 (4.1%) because of positive ANA and, of these 263 (40.9%) were evaluated by a rheumatologist. In 24/263 (9.1%) ANA-positive patients, the rheumatologist provided a diagnosis of SLE, while 39 (14.8%) had a diagnosis of another autoantibody-related rheumatic disease (AARD), 69 (26.2%) had no evidence of any disease, 29 (11%) had conditions that did not meet classification criteria for an AARD and the remainder (102, 38.8%) had a variety of rheumatologic diagnoses. The age range of the SLE patients was 25 to 73 years (mean 44.4 years), 95.8% were female, 87.5% were referred by a family physician and the average waiting time was 137.3 days. The serological profile of the 24 SLE patients included 29.1% anti-Sm, 25% anti-U1RNP, 25% anti-ribosomal P, 25% anti-SS-A/Ro60, 25% anti-Ro52/TRIM21, 8.3% anti-dsDNA but none were anti-DFS70-positive.

## Conclusions

This is the first study to evaluate the serological features of patients who were diagnosed on their first visit as having SLE after they were referred through a CT system because of positive ANA. Approximately 10% of the ANA referral patients were diagnosed as SLE and anti-Sm was the most common (~29%) autoantibody detected. This study provides an assessment of patients referred for positive ANA and implies that serological parameters might be helpful in determining the level of urgency of the referral.

